# Chikusetsu saponin IVa confers cardioprotection via SIRT1/ERK1/2 and Homer1a pathway

**DOI:** 10.1038/srep18123

**Published:** 2015-12-09

**Authors:** Jialin Duan, Ying Yin, Guo Wei, Jia Cui, Enhu Zhang, Yue Guan, Jiajia Yan, Chao Guo, Yanrong Zhu, Fei Mu, Yan Weng, Yanhua Wang, Xiaoxiao Wu, Miaomiao Xi, Aidong Wen

**Affiliations:** 1 Department of Pharmacy, Xijing Hospital, Fourth Military Medical University, Xi’an, 710032, China; 2 College of Pharmacy, Shaanxi University of Chinese Medicine, XianYang 712083, PR China

## Abstract

Hyperglycemia-induced reactive oxygen species (ROS) generation and Ca^2+^ overload contribute to the development of diabetic cardiomyopathy. In this study, we aimed to study the protective effects of Chikusetsu saponin IVa (CHS) from *Aralia taibaiensis* against hyperglycemia-induced myocardial injuries. Treatment of H9c2 cells with high glucose (HG) for 24 h resulted in a loss of cell viability and increase of ROS, LDH and Ca^2+^ levels, and also induced cell apoptosis, and those changes were all markedly reversed by the administration of CHS. In further studies, CHS dose-dependently increased the expression of Homer1a, ERK1/2 and SIRT1 in both H9c2 cells and rat primary cardiomyocytes. However, transfection of Homer1a-specific siRNA abolished the ability of CHS in controlling the ROS and Ca^2+^ homeostasis. Moreover, specific SIRT1 inhibitors or siRNA significantly suppressed the enhanced phosphorylation of ERK1/2 and expression of Homer1a induced by CHS as well as its cytoprotective effect. CHS induced Homer1a expression was also suppressed by siERK1/2. Additionally, results in diabetic mice also showed that CHS protected myocardium from I/R-introduced apoptosis by activating the SIRT1/ERK1/2/Homer1a pathway. These results demonstrated that CHS protected against hyperglycemia-induced myocardial injury through SIRT1/ERK1/2 and Homer1a pathway *in vivo* and *in vitro*.

Diabetic cardiomyopathy is one of the most common complications of diabetes and also a main cause of the mortality for diabetic patients who are at increased risk for developing cardiovascular diseases^1^,^2^. Hyperglycemia, a consequence of decreased glucose clearance and augmented hepatic gluconeogenesis, acts as a central role in the pathogenesis of diabetic cardiomyopathy. Many studies have demonstrated that increased production of reactive oxygen species (ROS) in diabetic cardiomyocytes, implicating hyperglycemia induces ROS production and oxidative damage in the heart, directly contribute to the development of diabetic cardiomyopathy^1^. Calcium is an important second messenger of various physiological processes. However, changes in levels of intracellular calcium can activate pathways that lead to apoptosis^3^. Intracellular calcium overload results in mitochondrial calcium accumulation, which facilitates the loss of mitochondria membrane potential and eventually induces the formation of ROS, and thus inducing the release of pro-apoptotic factors followed by apoptosis^4^,^5^,^6^,^7^. Given to the central role of the hyperglycemia in diabetic heart injury, preventing hyperglycemia-induced ROS and calcium overload are considered to be effective strategies in alleviating diabetic induced injury.

Homer proteins are commonly known as scaffold proteins at postsynaptic density and several different splice variants of the homer gene have been identified recently^8^. There are two different isoforms of Homer1 (Homer1a and Homer1 b/c), and Homer1a which is a short variant of Homer1, was the first Homer protein to be isolated^9^. Homer1a overexpression in neuronal cells attenuated mGluR-evoked intracellular calcium release, suggesting that Homer1a may regulate calcium release from the endoplasmic reticulum (ER)^10^. Recent studies showed that Homer1a expressed in the heart as well as in the brain and played an important role in cardiac hypertrophy^11^. However, the role of Homer1a in diabetic cardiomyopathy has not previously been determined.

Mammals have seven sirtuins paralogs (SIRT1-7), more recent works have implicated that sirtuins can modulate a variety of biological processes including autophagy, growth suppression, apoptosis, transcriptional silencing, inflammation, metabolism, and stress response^12^,^13^,^14^,^15^. SIRT1, the best-characterized sirtuin among the seven, seems to have evolved to respond to a variety of stresses and emerged as the key antiaging molecule and regulator in many diseases, including type II diabetes and cardiovascular disease^16^. Several studies have demonstrated that SIRT1 overexpression positively affects the MAPK pathway in the ischemia/reperfusion heart^17^. Activation of the ERK1/2 signaling pathway has also been shown to regulate induction of Homer1a in other cell types^18^,^19^, however, the relationship between SIRT1/ERK1/2 pathway and Homer1a in diabetic cardiomyopathy is still unclear.

Saponin has a high potential for antidiabetic remedy, and the hypotriglyceridemic and hypocholesterolemic actions of saponin can help diabetic patients in reducing the risk of atherosclerosis^20^. Chikusetsu saponin IVa (CHS) is the most powerful antioxidant among the triterpenoid saponins isolated from the Chinese herb *Aralia taibaiensis*. Saponins of *Aralia taibaiensis* could efficiently attenuate high glucose (HG)-induced cardiomyocytes apoptosis by stimulating the production of Nrf2-regulated antioxidant enzymes in cardiomyocytes^21^. And further study indicated CHS pretreatment could protect the brain from cerebral ischemia in diabetes stroke models^22^. In our recent study, we also found that CHS improved cardiac function in mice with diabetic cardiac disorders (data not shown). Nevertheless, the underlying mechanisms through which CHS protected against hyperglycemia-induced oxidative stress and Ca^2+^ overload in cardiomyocytes were still not known. The present study was designed to elucidate whether CHS protected against hyperglycemia-induced ROS explosion and Ca^2+^ overload by stimulating the expression of Homer1a and the involvement of SIRT1/ERK1/2 signaling *in vivo* and *in vitro*.

## Results

### CHS ameliorated HG-induced cytotoxicity and cardiomyocytes apoptosis

**Figure 1:**
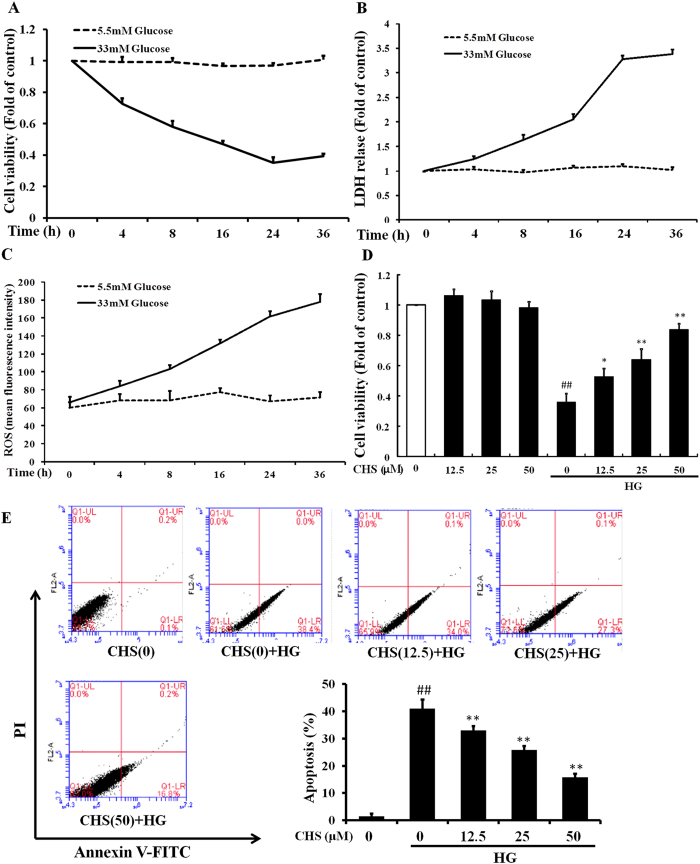
Cytoprotective effects of CHS on HG-induced H9c2 cell injury. H9c2 cells were treated with 5.5 or 33 mM glucose for 36 h, then, cell viability (**A**), LDH (**B**) and ROS (**C**) were detected at 4 h, 8 h, 16 h, 24 h and 36 h, separately. D. H9c2 cells were pretreated with various concentrations of CHS (12.5, 25 and 50 μM) for 24 h, and then exposed to HG (33 mM) for another 24 h. Cell viability was monitored by the MTT assay. E. Inhibition of HG-induced cell apoptosis by CHS was detected by flow cytometry using annexin V-FITC and PI. ^*##*^*P* < 0.01 vs control group, ^**^P < 0.01 vs model group.

HG induced a time-dependent cytotoxic effect on H9c2 cells as expected. Cells were treated with 5.5 or 33 mM glucose for 36 h, then, cell viability, ROS and LDH levels were detected at 4 h, 8 h, 16 h, 24 h and 36 h, respectively. Assessment of cell viability and LDH release indicated that HG induced a time-dependent cytotoxic effect on H9c2 cells (Fig. 1A,B). In addition, HG significantly increased the production of ROS in cells in a time-dependent manner (Fig. 1C). To investigate the protective effect of CHS on HG-induced cell injury, cell viability and apoptosis were tested. H9c2 cells were pretreated with various concentrations of CHS (12.5, 25 and 50 μM) for 24 h before being exposed to HG for another 24 h. Cell viability was monitored by the MTT assay, and representative dose–response viability data were shown in Fig. 1D. This was accompanied by a decrease of apoptotic rates. Both the ability of HG to induce apoptosis in H9c2 cells and the antiapoptotic role of CHS were determined by the Annexin V and PI staining assay. CHS treatment decreased the apoptotic population in a dose-dependent manner (Fig. 1E). These results revealed a cytoprotective role of CHS.

### CHS inhibited HG-induced Ca^2+^ and ROS accumulation

**Figure 2:**
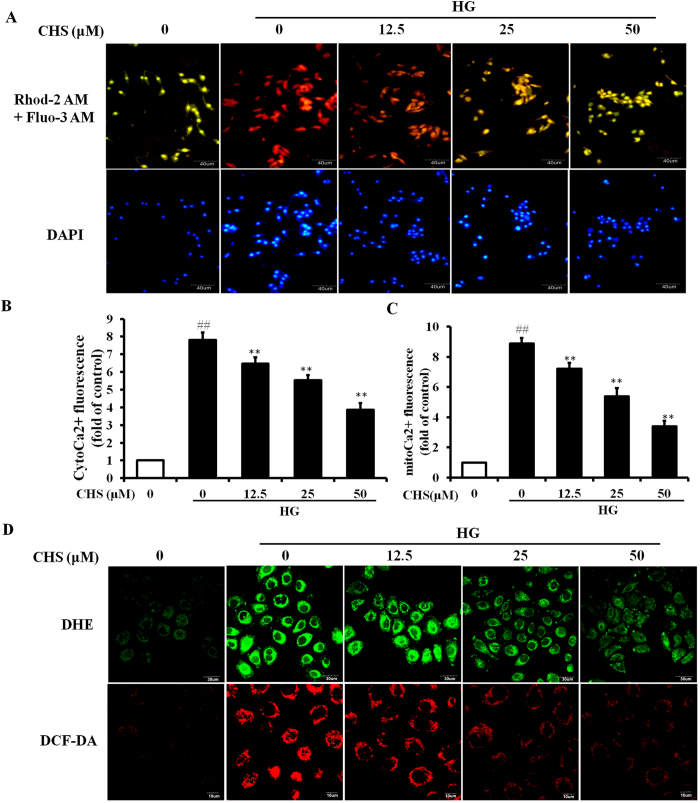
Effects of CHS on HG-induced calcium overload and ROS accumulation in H9c2 cells. (**A**) H9c2 cells were pretreated with various concentrations of CHS (12.5, 25 and 50 μM) for 24 h, and then exposed to HG for another 24 h. The representative staining for Fluo3-AM (green signal), Rhod-2 AM (red signal) and DAPI (blue signal) were shown by confocal microscopy (40×). (**B,C**) were representative line graph depicts percent of fluorescent positive cells detected by flow cytometry upon staining with fluorescent dyes Fluo-3 AM (Ex/Em 488/525) specific for cytosolic calcium and Rhod-2 AM (Ex/Em 488/590) specific for mitochondrial calcium after CHS treatment CHS. D. The intracellular ROS levels were measured in terms of O2•− and H2O2 using DHE and DCF-DA, respectively. ^*##*^*P* < 0.01 vs control group, ^**^P < 0.01 vs model group.

Since mitochondria act as intracellular calcium stores, Rhod-2 AM, the probe specific for intra-mitochondrial calcium, was used in conjunction with Fluo-3 AM. Using these dyes, calcium localization was determinated by confocal microscopy. Exposure of H9c2 cells to HG resulted in an increase in the fluorescent intensity of the Fluo-3 AM and Rhod-2 AM positive cells. This indicated that the cytosolic calcium increased and accumulated in the mitochondria as a result of HG treatment (Fig. 2A). Pretreating cells with CHS for 24 h abolished most of the increase in mitochondria calcium accumulation caused by HG. Quantitative data from flow cytometry were matched with the results from confocal imaging. The quantitative estimation for intracellular and mitochondria calcium levels, indicated by percent-fluorescent positive population of Fluo-3 AM and Rhod-2 AM stained cells. Under the HG condition, the percent of fluorescent positive population of Fluo-3 AM and Rhod-2 AM stained cells increased remarkably. When cells were pretreated with CHS, the increases of calcium were almost inhibited (Fig. 2B,C).

Calcium homeostasis is closely coupled to ROS accumulation. Thus ROS increase is likely to play an important role in Ca^2+^ regulation and excessive ROS may contribute to calcium dysregulation. Therefore, we measured intracellular levels of ROS in HG-treated cells pretreated with CHS. The intracellular ROS levels were measured in terms of O_2_•− and H_2_O_2_ using DHE and DCF-DA, respectively. Exposure of H9c2 to HG led to an increase in DHE and DCF fluorescence as compared to control cells. The increase in DHE and DCF fluorescence upon exposure to HG was eliminated by CHS. These results showed that O_2_•− and H_2_O_2_ were dramatically increased in HG-treated cells, and CHS was able to abrogate HG-induced ROS accumulation (Fig. 2D).

### CHS ameliorated HG-induced apoptosis

**Figure 3:**
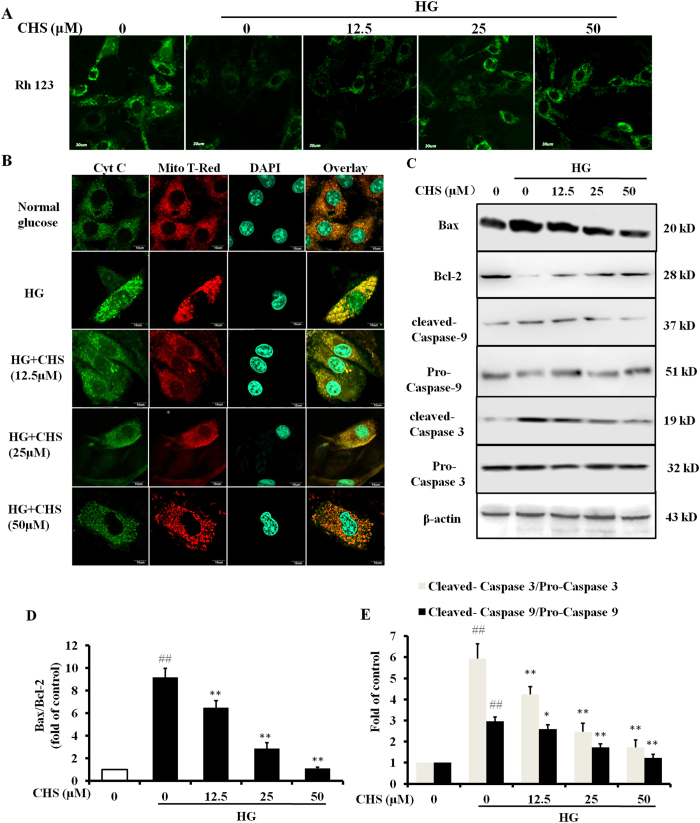
Effects of CHS on HG-induced apoptosis in H9c2 cells. (**A**) H9c2 cells were pretreated with various concentrations of CHS (12.5, 25 and 50 μM) for 24 h, and then exposed to HG for another 24 h. Mitochondrial membrane potential was measured by rhodamine 123 (Rh 123). (**B**) Effects of CHS on HG-induced cytochrome c release from mitochondria. (**C**) Effects of CHS on HG-induced caspase-9, caspase-3, Bax and Bcl-2 expressions in H9c2 cells. (**D,E**). Quantification histograms indicated changes between CHS and HG groups. ^*##*^*P* < 0.01 vs control group, ^**^P < 0.01 vs model group.

Since the increase in intracellular Ca^2+^ and excessive ROS lead to mitochondrial dysfunction associated with apoptosis, next we tested the effect of CHS on HG-introduced loss of mitochondrial membrane potential. Treatment of cardiomyocytes with HG for 24 h resulted in a decrease in Rh123 fluorescence as detected by confocal microscopy when compared to control cells, indicating HG-induced mitochondria dysfunction in H9c2 cells (Fig. 3A). The decrease in Rh123 fluorescence upon treatment with HG was restored by CHS. We also determined HG-induced cytochrome c release from mitochondria. In normal cells, cytochrome c immunoreactivity was co-localized in the mitochondria (Fig. 3B). After exposure of H9c2 cells to HG, the cells exhibited a diffuse distribution of cytochrome c immunoreactivity throughout the cytoplasm and a decrease in mitochondria-associated cytochrome c, indicating that there was a significant release of cytochrome c from the mitochondria to the cytoplasm. Pretreatment with CHS abolished this phenomenon efficiently.

Increasing evidence indicates that the release of cytochrome c into the cytosol, where it forms a complex with Apaf-1 and caspase-9, and then results in caspase-9 activation. Caspase-9 then cleaves and activates caspase-3 which plays an important role in the execution of apoptosis in a number of different cell types. Immunoblot analysis indicated that HG treatment induced elevation of Bax/Bcl-2 ratio, cleavage of caspase-9 and caspase-3, and exposure of the cells to CHS for 24 h was able to inhibit these activities (Fig. 3C–E). These results together suggested that CHS might be able to inhibit HG-induced apoptosis associated with mitochondria dysfunction.

### CHS ameliorated HG-induced Ca^2+^ accumulation via induction of Homer1a

**Figure 4:**
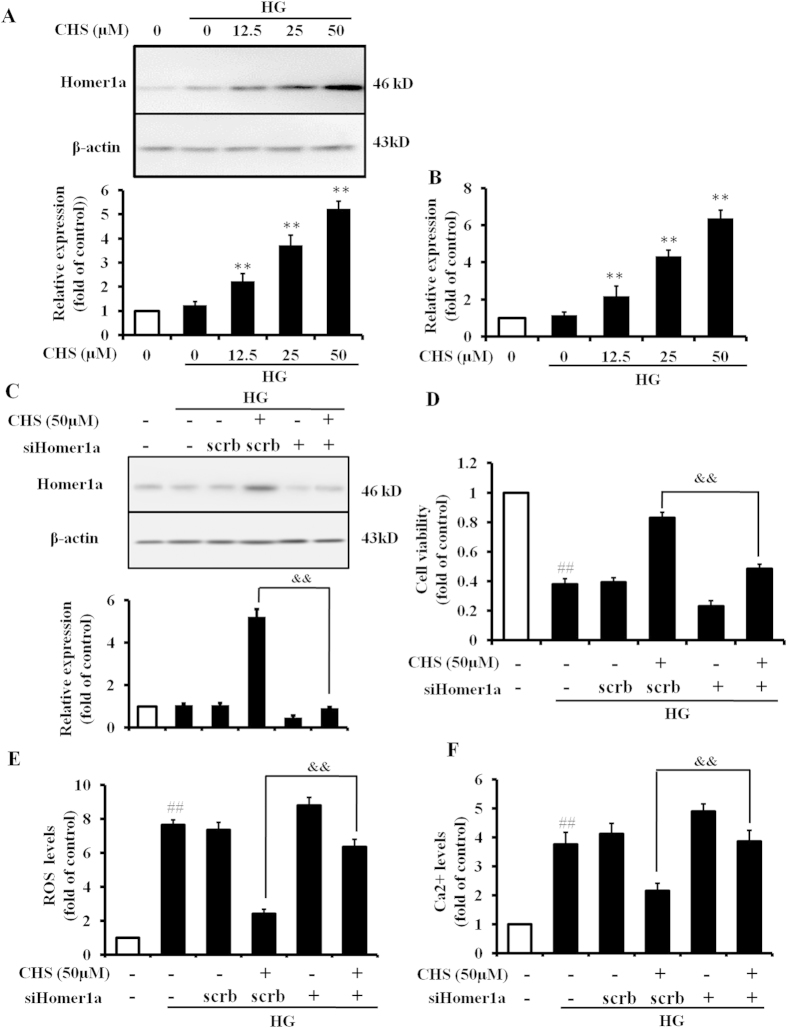
Involvement of the Homer1a in cardioprotection by CHS in H9c2 cells. (**A**) Immunoblot and (**B**) real time PCR analysis showed the protein and mRNA expression of Homer1a in H9c2 cells pretreated with CHS for 24 h before administration of HG. (**C**) After transfection of Homer1a-specific siRNA, H9c2 cells were pretreated with CHS, and then subjected to HG. Homer1a level was determined by western blot analysis. (**D**) Cell viability was monitored by the MTT assay. (**E**) ROS was detected by flow cytometry using DCF-DA. (**F**) Ca^2+^ levels in H9c2 detected by flow cytometry upon staining with fluorescent dyes Fluo-3 AM. ^*##*^*P* < 0.01 vs control group, ^**^P < 0.01 vs model group, ^*&&*^*P* < 0.01 vs CHS plus scrb group.

Homer1a has been implicated to regulate the intracellular Ca^2+^ homeostasis. To assess the relationship between Ca^2+^ homeostasis and Homer1a, cells were pretreated with CHS for 24h, and then subjected to HG. We first sought to determine whether CHS had an effect on Homer1a expression. The results from western blot analysis indicated that treatment of cells with HG for 24 h slightly increased Homer1a protein levels compared with control group. However, pretreatments with CHS significantly increased Homer1a protein levels compared with HG group (Fig. 4A). Additionally, results obtained from real time PCR revealed that CHS significantly increased the expression of Homer1a mRNA (Fig. 4B). These results were consistent with the results from western blot.

To confirm the importance of Homer1a in CHS-mediated cardioprotection, we used siRNA for Homer1a knockdown. After siRNA transfection, the groups were either treated or not treated with CHS, and then cells were exposed to HG for 24 h. As shown in Fig. 4C, siHomer1a significantly inhibited Homer1a expression. In Homer1a knockdown cells, CHS could not restore cell viability (Fig. 4D). In addition, siRNA also abolished the ability of CHS in controlling the ROS and Ca^2+^ homeostasis (Fig. 4E,F). These results suggested that Homer1a was an essential mediator of CHS against HG-induced oxidative stress and Ca^2+^ overload.

### CHS-induced Homer1a upregulation is mediated by the ERK1/2 pathway

**Figure 5:**
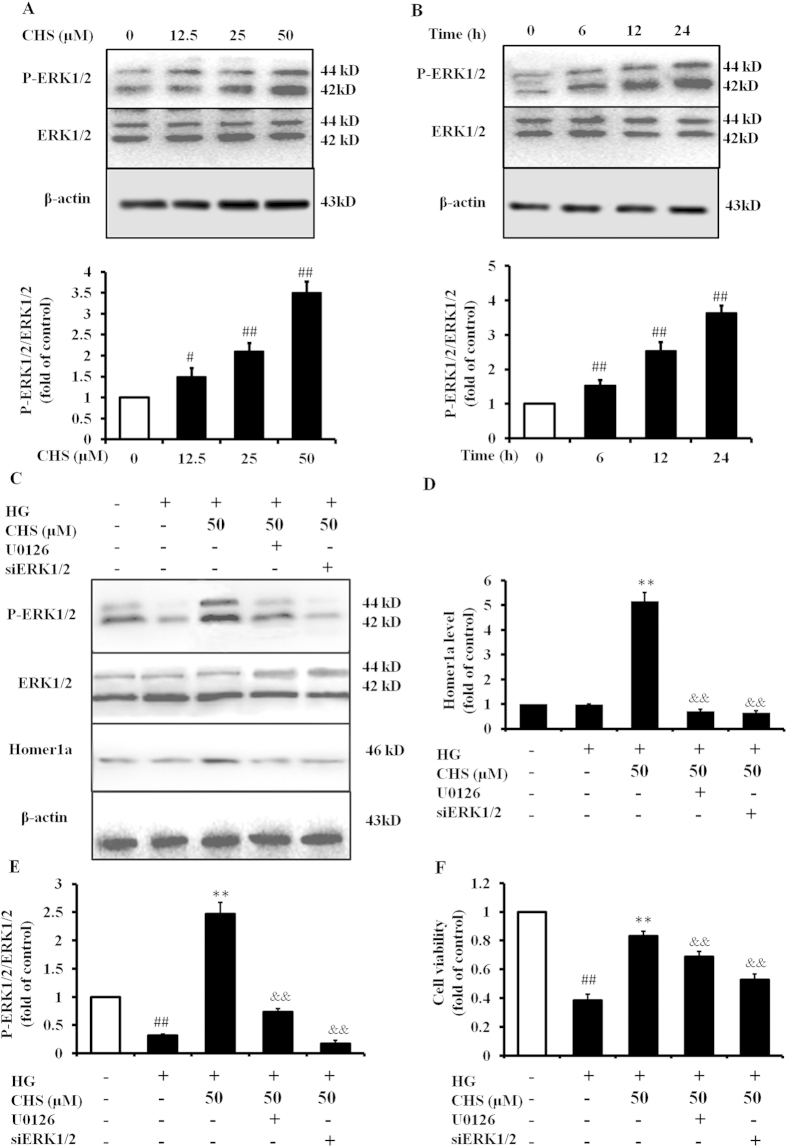
Involvement of ERK1/2 in the activation of Homer1a by CHS. (**A**) H9c2 cells were cultured in normal glucose medium (5.5 mM) with different concentrations of CHS (12.5, 25 or 50 μM) for 24 h. Then, the cells were harvested and protein level was determined by western blot. (**B**) H9c2 cells were incubated with CHS (50 μM) for different periods (0–24 h) as indicated. ^*##*^*P* < 0.01 vs 0 group. (**C**) H9c2 cells were pretreated with CHS (50 μM) combined with or without U0126 (10 μM) or siERK1/2, then incubated with high glucose (33 mM). Homer1a and P-ERK1/2 protein levels were determined by Western blot. Quantification histograms of Homer1a and P-ERK1/2 were showed in D and E. F. Cell viability was monitored by the MTT assay. ^*##*^*P* < 0.01 vs control group, ^**^P < 0.01 vs model group. ^*&&*^*P* < 0.01 vs CHS group.

Previous studies have reported that ERK1/2 signaling is implicated in Homer1a mRNA induction. To elucidate the upstream signaling event leading to activation of Homer1a, we further defined the roles of ERK1/2 signaling cascades in Homer1a mRNA induction. We first monitored the expression of expression of phosphorylated ERK1/2 (p-ERK1/2) by immune blotting at the indicated times after CHS treatment. As shown in Fig. 5A,B, western blot analysis of cell lysate demonstrated significant augmentation of ERK1/2 phosphorylation in concentration- and time-dependent manners. To further confirm the effects of ERK1/2 on the Homer1a expression induced by CHS, ERK1/2 inhibitor U126 and siERK1/2 were used. Expression of p-ERK1/2/ERK1/2 was significantly increased in cells preconditioned with CHS in comparison to the HG group (Fig. 5C). Data from the groups pretreated cells with the highly selective ERK1/2 inhibitor and siRNA indicated that ERK1/2 inhibition abolished CHS-induced Homer1a expression (Fig. 5C,D), blocked the protective effect of CHS (Fig. 5F). These results suggested that ERK1/2 might be involved in mechanisms of CHS-stimulated Homer1a induction.

### SIRT1 is involved in the activation of ERK1/2 induced by CHS

**Figure 6:**
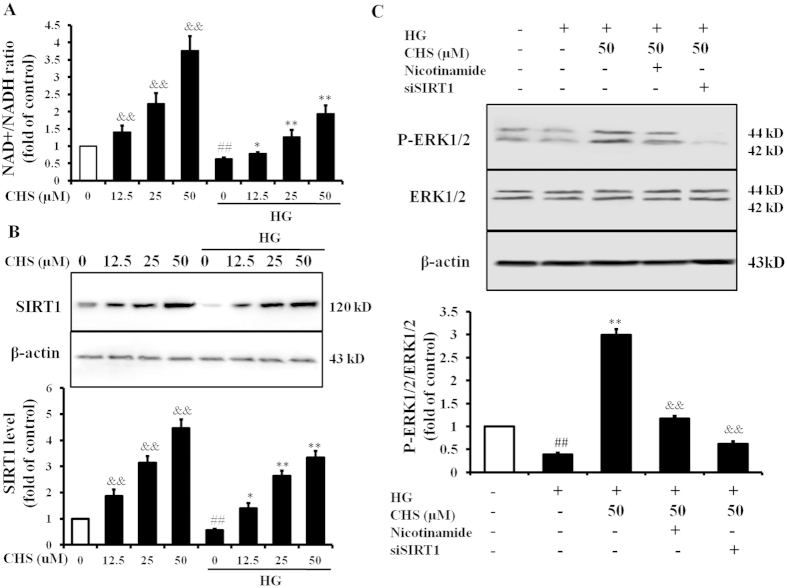
Involvement of SIRT1 in the activation of ERK1/2 by CHS. (**A**) H9c2 cells were pretreated with CHS only or then incubated with high glucose (33 mM). NAD+ and NADH levels were measured by a kit, then NAD+/NADH was calculated. ^*##*^*P* < 0.01 vs 0 group without HG, ^*&&*^*P* < 0.01 vs group treated with nothing, ^**^P < 0.01 vs 0 group treated with HG. (**B**) Immunoblot analysis showed the protein expression of Homer1a in H9c2 cells pretreated with CHS with or without HG. ^*##*^*P* < 0.01 vs 0 group without HG, ^*&&*^*P* < 0.01 vs group treated with nothing, ^**^P < 0.01 vs 0 group treated with HG. (**C**) After transfection of Homer1a-specific siRNA or treatment with nicotinamide (a SIRT1 inhibitor, 200 nM), H9c2 cells were pretreated with CHS, and then subjected to HG. ERK1/2 phosphorylation level was determined by western blot analysis. ^*##*^*P* < 0.01 vs control group, ^**^P < 0.01 vs model group. ^*&&*^*P* < 0.01 vs CHS group.

It has been reported that SIRT1 modulated MAPK pathways in an experimental model of IR using cardiomyocytes^17^. To explore the effect of CHS on SIRT1 expression and relationships between SIRT1 and ERK1/2 in HG-treated cardiomyocytes, the protein levels of SIRT1 and ERK1/2 were assessed by immunoblotting. The results showed that the level of NAD+ and the expression of SIRT1 in cells were increased after incubation with CHS for 24 h in a concentration-dependent manner (Fig. 6A,B). Further, we explored whether SIRT1 is also involved in the ERK1/2 pathway. For this purpose, the SIRT1 inhibitor nicotinamide and siRNA were used. The phosphorylation of ERK1/2 was increased by CHS, but attenuated by pre-treatment with nicotinamide or siSIRT1 (Fig. 6C). All the results showed that CHS could upregulate SIRT1 which was involved in the activation of ERK1/2.

### CHS attenuated HG-induced apoptosis by activating the SIRT1/ERK1/2/Homer1a pathway

**Figure 7:**
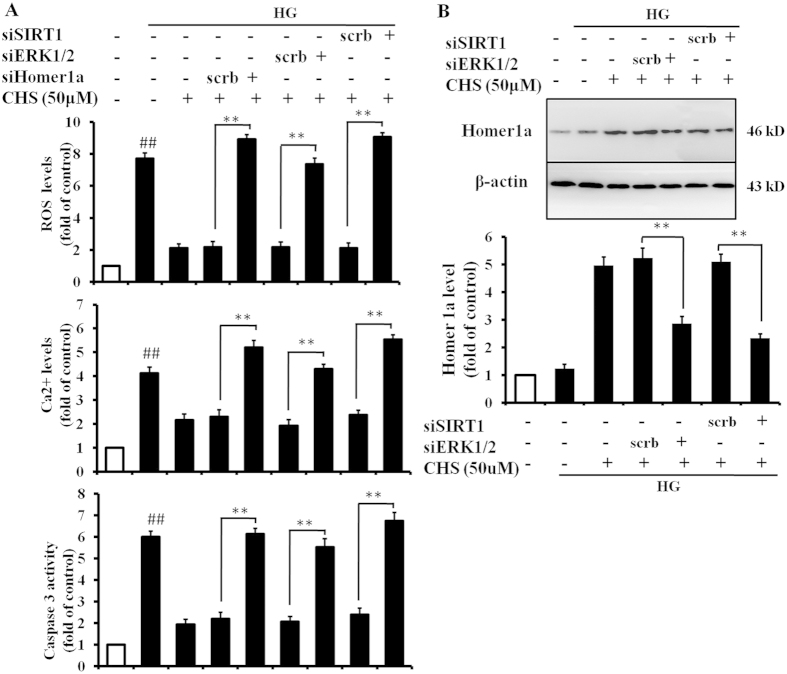
Involvement of SIRT1/ERK1/2/Homer1a pathway in the protective role of CHS. H9c2 cells were transfected with SIRT1, Homer1a and ERK1/2 siRNA followed by treatment with CHS and high glucose. (**A**) The intracellular ROS level was measured by DCF-DA using flow cytometry using DCF-DA. Ca^2+^ level was detected by flow cytometry upon staining with fluorescent dyes Fluo-3 AM. Caspase 3 levels were measured by a kit. B. After transfection with SIRT1 and ERK1/2 siRNA, H9c2 cells were pretreated with CHS, and then subjected to HG. Homer1a level was determined by Western blot analysis. ^*##*^*P* < 0.01 vs control group, ^**^P < 0.01 vs scrb group.

To further study the role of SIRT1/ERK1/2 and Homer1a signaling pathways in CHS-mediated suppression of apoptosis induced by HG, apoptosis rate, ROS and Ca^2+^ levels were tested. In this study, cells transected with siSIRT1, siERK1/2 and siHomer1a exhibited abnormal levels of ROS, Ca^2+^ and caspase 3 relative to cells that had been treated with CHS alone, indicating that the protective effect of CHS was largely counteracted by SIRT1, ERK1/2 and Homer1a siRNA (Fig. 7). In addition, siSIRT1 and siERK1/2 also reduced the level of Homer1a compared with CHS-treated group. These results showed that CHS suppressed HG-induced apoptosis by activating the SIRT1/ERK1/2/Homer1a pathway in H9c2 cells.

### Suppression of apoptosis by CHS is mediated through SIRT1/ERK1/2-dependent Homer1a activation in primary cardiomyocytes exposed to HG

**Figure 8:**
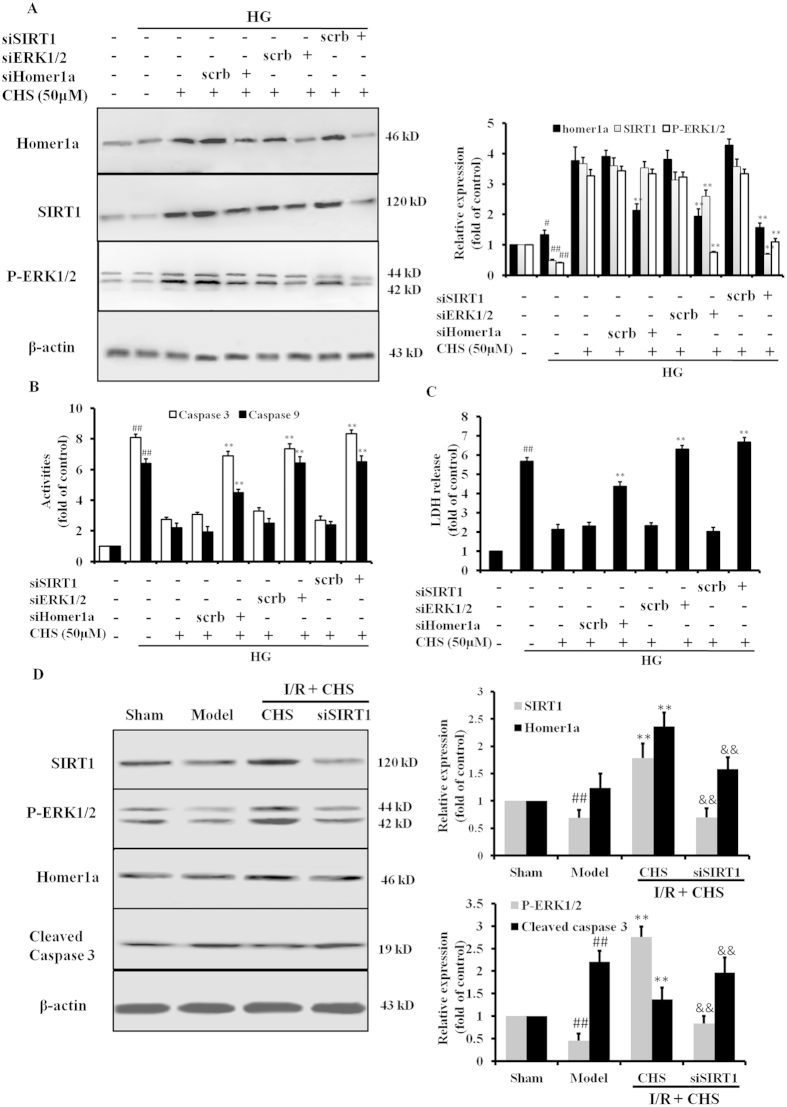
Suppression of apoptosis by CHS was mediated through SIRT1/ERK1/2-dependent Homer1a activation in primary cardiomyocytes. (**A**) Cultured cardiomyocytes were incubated with CHS (50 μM) and high glucose (33 mM) with or without siSIRT1 or siERK1/2 or siHomer1a. The non-specific scramble siRNA (scrb) was used as a negative control. A. Protein levels were analyzed by western blot. (**B**) Caspase 3 and caspase 9 were determined by appropriate kits. (**C**) LDH release was measured by a kit. ^*##*^*P* < 0.01 vs control group, ^**^P < 0.01 vs scrb group. (**D**) Suppression of I/R-induced cardiac apoptosis of CHS by Homer1a-dependent antioxidant activities is through SIRT1/ERK1/2 signaling activation in STZ-triggered diabetic mice. ^*##*^*P* < 0.01 vs sham group, ^**^P < 0.01 vs model group, ^*&&*^*P* < 0.01 vs CHS group.

To further confirm the experimental results from H9c2 cells, primary cardiomyocytes were used in the following experiments. Isolated cardiomyocytes from neonatal rat hearts were grown in media containing 33 mM glucose and CHS, together with siSIRT1, siERK1/2 or siHomer1a. The results of the western blot assay showed that enhancement of CHS on SIRT1, ERK1/2 and Homer1a protein expressions were suppressed by the treatment of SIRT1-specific siRNA (Fig. 8A), which also reversed the inhibition of CHS on HG-induced cleaved caspase 3 levels. And siERK1/2 had no effect on the SIRT1 expression, but suppressed P-ERK1/2 and Homer1a protein expressions. These findings indicated that CHS protected against cardiac apoptosis by activating the SIRT1/ERK1/2/Homer1a pathway.

In order to determine whether the SIRT1/ERK1/2 and Homer1a pathways were activated after administration of CHS *in vivo*, the protein levels of SIRT1, ERK1/2 and Homer1a in rat left ventricular tissue were evaluated by immunoblot analysis. We found that I/R resulted in a dramatic decrease in protein levels of SIRT1 and P-ERK1/2 and an increase in cleaved-caspase 3 levels in diabetic hearts. However, the levels of those proteins were significantly reversed in cardiac cells of mice receiving CHS (Fig. 8D).

## Discussion

Diabetic cardiomyopathy, as an early complication of diabetes, is manifested by diastolic dysfunction followed by abnormalities in systolic function^23^. Hyperglycemia-induced apoptosis in cardiomyocytes is intimately linked to diabetic complications^24^. Both, oxidative stress and elevation of calcium concentration have been implicated as important mediators in the effectors phase in hyperglycemia-induced cardiac cell apoptosis^25^,^26^. Hyperglycemia-induced oxidative stress is a major risk factor for the development of micro-vascular pathogenesis in the diabetic myocardium, which results in myocardial cell death, fibrosis, hypertrophy, abnormalities of calcium homeostasis, and endothelial dysfunction^27^,^28^. Large experimental and clinical studies have shown that the generation of ROS is increased in diabetic cardiomyopathy, and closely associated with oxidative stress^29^. Hyperglycemia has been shown to activate the calcium channels of cardiac myocytes and cause an acute rise of intracellular calcium concentration^30^. Elevated glucose induced calcium mediated activation of caspase-12, caspase-9 and caspase-3 in the cardiomyocytes, and further caused myocardial apoptosis in the mitochondria-dependent and -independent pathways^31^,^32^.

In the present study, cardiomyocytes subjected to simulated HG, exhibited marked biochemical changes, including decreased cell vitality, increased ROS production and cell death. As the results showed, CHS reduced HG-induced cytotoxicity and cardiomyocytes apoptosis effectively, which was confirmed by three different methods including MTT, annexinV-FITC/PI staining, and LDH release. It is well established that mitochondria Ca^2+^ accumulate during the elevation of cytosolic Ca^2+^ in cardiomyocytes subjected to HG. Our calcium imaging data showed that HG initially increased the intracellular and cytosolic calcium concentration in H9c2 cells subjected to HG for 24 h. However, cells pretreated with CHS showed much lower fluorescence intensity in mitochondria and cytosolic than that in HG group. These results were proven by flow cytometry. There is accumulating evidence showing that calcium leads to the generation of ROS, such as hydroxyl radicals (.OH), O_2_^-^, and H_2_O_2_^33^. In order to make clear whether CHS had some effects on HG-induced oxygen free radical production, H9c2 were stained with DHE and DCF-DA. Data from confocal imaging showed that CHS decreased DHE and DCF fluorescence, and these results suggested that CHS abrogated HG-induced ROS and calcium accumulation.

Studies have shown that mitochondria, as a source of ROS, and a sensor of oxidative stress, play a key role in the transduction and amplification of the apoptotic response in cardiomyocytes during oxidative stress^34^. ROS promote the release of cytochrome c by increasing mitochondrial permeability. Pro-caspase-3 is downstream of cytochrome c in the apoptotic cascade and is cleaved by active caspase-9 to produce active caspase-3, which contributes to oligonucleosomal DNA fragmentation, and ultimately leads to apoptosis^35^,^36^. In the heart under physiological condition, Ca^2+^ plays an important role in mitochondrial oxidative-phosphorylation which enhances ATP production to support contractile activity and ion transport systems of myocardium^37^. However, cytosolic Ca^2+^ increases abnormally in cardiomyocytes as a consequence of I/R or other stresses, causing myocardial apoptosis and cell death. Also HG induces Ca^2+^ overload which promotes oxidative stress and ultimately leads to cell apoptosis. These two affronts are linked to each other, viz., the oxidative stress induces [Ca^2+^] i and increase in [Ca^2+^] i induces oxidative stress^38^. We found that CHS inhibited HG-induced activation of caspase-9 and caspase-3, reduced cytochrome c release from the mitochondria to the cytoplasm, and improved HG-induced impairment of the mitochondrial membrane potential. The present study clearly demonstrated that CHS inhibited HG-induced apoptosis by blocking Ca^2+^-dependent and mitochondria-dependent apoptosis.

Homer proteins, including Homer1, Homer2 and Homer3, are more recently discovered postsynaptic scaffolding proteins that play a central role in Ca^2+^ signaling^9^. Homer1 proteins have been shown to be associated with many Ca^2+^ signaling proteins, including IP_3_ receptors (IP_3_Rs), G protein-coupled receptors, canonical transient receptor potential (TRPC) channels and notably, the ryanodine receptors (RyRs)^39^,^40^,^41^,^42^. Homer1a is an immediate early gene, while Homer1b/c is constitutively expressed, and Homer1a may act in a dominant negative fashion by interfering with multimerization and disassembling signaling complexes^43^,^44^,^45^. Westhoff reported that Homer1c decreased the RyR2-mediated Ca^2+^ release from microsomes prepared from the sarcoplasmic reticulum of rat cardiac myocytes. However, Homer1a reversed the effect of Homer1c, indicating that Homer proteins might regulate the release of Ca^2+^ from intracellular stores in cardiac myocytes^46^. Previous studies had shown that Homer1a might act as a Ca^2+^-dependent endogenous ROS scavenger, and such anti-oxidative feature would play an essential role in improving mitochondrial function^47^. Thus, we hypothesized that Homer1a protein might be also involved in the cardioprotective effect of CHS preconditioning on HG damage. Interestingly, CHS induced expression of Homer1a in the protein and gene levels in the cells subjected to HG. We also found that inhibition of Homer1a by siRNA almost completely blocked the protective of CHS in HG-induced oxidative stress and Ca^2+^ overload.

We further demonstrated that the SIRT1/ERK1/2 pathway was essential for the activation of Homer1a by CHS in H9c2 cell. The SIRT1, a NAD-dependent histone deacetylase, has emerged as a critical regulator in response to oxidative stress^48^. It expressed in all organs of the body including the heart, brain, pancreas, liver, spleen, skeletal muscle, and adipose tissues. In cardiomyocytes, SIRT1 is primarily localized in the nucleus during embryonic period, but in myocytes of adult heart, it is dominantly expressed in the cytoplasm^49^. Increasing evidence has shown that SIRT1 is closely related to cardiovascular diseases and regulates a number of key physiological and metabolic processes including stress resistance, senescence, apoptosis and energy metabolism^50^. ROS produced during oxidative stress could activate mitogen-activated protein kinases (MAPKs), a group of apoptotic signaling regulatory proteins, which mediate cellular responses^51^. Several studies have showed that the suppression of ERK1/2 and activation of JNK and p38 MAPKs play a role in apoptosis in different cell lines^52^. A variety of stress stimuli, including oxidative stress, induces functional changes of these kinases. Recently, several works implied that ERK1/2 cascaded plays a key role in Homer1a mRNA expression in cultured cortex neurons^53^, and some works reported that MAPK pathways might affect the expression of Homer 1a in cardiomyocytes^54^. As mentioned above, the possible beneficial effects of CHS on cardiomyocytes subjected to HG might closely relate to Homer1a expression. To clarify these effects, SIRT1 and ERK1/2 were studied, and we hypothesized the mechanism is that SIRT1 activation induced up-regulation of ERK1/2, which leads to the expression of Homer1a.

Our results clearly demonstrated that CHS pretreatment significantly increased ERK1/2 phosphorylation in cardiomyocytes in concentration- and time-dependent manners. We also found that inhibition of ERK1/2 with the specific inhibitor or ERK1/2 siRNA abolished the protective effect of CHS and upregulation of Homer1a. These findings suggested that the involvement and interaction between the ERK1/2 and Homer1a pathways play an indispensable role in the underlying mechanisms of cardioprotection mediated by CHS. It has been reported that SIRT1 has an effective role in protecting cardiac cells from I/R injury and the involvement of this protein in the modulation of MAPKs^23^. In the present study, we found that CHS increased the level of NAD+ and the expression of SIRT1. In addition, specific SIRT1 inhibitors or siRNA significantly suppressed the enhanced phosphorylation of ERK1/2 and expression of Homer1a induced by CHS as well as its cytoprotective effect. Collectively, based on our results, it was suggested that CHS exerted its cardiac protective effect through the activation of signaling pathways mediated by SIRT1 and ERK1/2/Homer1a. In addition, results in neonatal primary cardiomyocytes and diabetic mice also showed that CHS protected myocardial cells from HG-induced apoptosis by activating the SIRT1/ERK1/2/Homer1a pathway.

In summary and conclusion, we demonstrated that CHS protected cardiomyocytes from HG-triggered oxidative stress and calcium overload in H9c2 cells, neonatal primary cardiomyocytes and diabetic mice. The underlying mechanisms of CHS-mediated cardioprotection might be attributable to activation and cross-talk between the SIRT1/ERK1/2/Homer1a pathways. These results supported further investigation of CHS as a promising novel therapeutic agent for diabetic cardiomyopathy.

## Materials and Methods

### Animals

The experimental protocol was approved by the Ethics Committee for Animal Experimentation and was performed according to the Guidelines for Animal Experimentation of the Fourth Military Medical University and the National Institute of Health Guide for the Care and Use of Laboratory Animals (NIH Publications No. 80–23) revised in 1996. The mice were provided by the Experimental Animal Center of the Fourth Military Medical University. The animals were housed under a 12-h light–dark cycle and temperature was kept at 25 °C.

### Type 2 diabetic mouse model

Diabetes mellitus was induced in male C57BL/6 mice 8–12 weeks old, weighing 23–25 g, by intraperitoneal injection of streptozotocin (STZ, Sigma, St Louis, MO, USA) at a dose of 50 mg/kg dissolved in 100 mM citrate buffer pH 4.5 for five consecutive days. Control animals were injected with the same volume of vehicle. After 4 weeks, blood glucose levels were measured using Bayer’s BREEZE2 meter (Bayer Health Care LLC, Mishawaka, USA) by tail vein blood sampling. Mice with blood glucose levels of >11.1 mM were used for the present study. The animals were exposed to 30 min ischemia followed by 24 h of reperfusion.

### Cell culture and treatment

Rat H9c2 myoblasts (American Type Culture Collection, ATCC, Maryland, USA) were grown in Dulbecco’s modified Eagle’s medium (DMEM, Life Technologies, Grand Island, USA) supplemented with 5.5 mM glucose, 10% fetal bovine serum (FBS, Life Technologies, Grand Island, USA) and 1% antibiotics (penicillin/streptomycin, Sigma, St. Louis, MO). The medium was changed every 2–3 days. One day before the experiments, cells were seeded in 96-well culture dishes (10^4^ per well) or 6-well culture dishes (10^6^ per well). In all experiments, the cells were pretreated with different concentrations of CHS with or without different inhibitors for 24 h before the addition of glucose at the final concentration of 33 mM for 24 h. All experiments were repeated three times for each treatment condition in each experiment.

### Primary culture of neonatal rat cardiomyocytes

Neonatal rat cardiomyocytes were prepared from the ventricular tissue of 1- to 3-days old Sprague-Dawley rats by a method described by Fujio Y^55^. Ventricles from ten rats were aseptically removed and minced into approximately 1 mm^3^ chunks in serum-free PBS balanced salt solution. The cardiomyocytes were disaggregated by repeated digestion with 0.125% collagenase I (Sigma, St. Louis, MO) dissolved in PBS at 37 °C for 6 times. The supernatants were collected into centrifuge glass tube containing warm DMEM supplemented with 10% FBS. Then cells were gently sedimented at 800 g for 5 min. The cells were resuspended in DMEM supplemented with 10% FBS, and cultured in a 10-cm diameter plastic Petri dish for 90 min in 5% CO_2_ at 37 °C. The culture suspension was carefully removed and seeded into a six-well plate. The cells were incubated at 37 °C in 5% CO_2_ incubator overnight, and the medium was changed every other day. Then cells were treated as the required.

### Cell viability

Viability of cardiomyocytes was determined by 3-[4, 5-Dimethylthiazol-2-yl]-2, 5-diphenyltetrazolium bromide (MTT, St. Louis, MO) assay. Cells were seeded at 1 × 10^4^ cells/well in 96-well plates. After different treatment, 20 μl of 5 mg/ml MTT solution was added to each well (0.1 mg/well), and wells were incubated for 4 h at 37 °C. The supernatants were aspirated, the formazan crystals in each well were dissolved with 150 μl of DMSO, and optical density at 490 nm was read on a Microplate Reader (Tecan, Maennendorf, Switzerland).

### Lactate dehydrogenase (LDH) assay

The release of LDH was used to determine the cytotoxicity by a diagnostic kit according to the manufacturer’s instructions (Nanjing Jiancheng Bioengineering Institute, Nanjing, P.R. China). After different treatments, 50 μL of supernatant from each well was collected and incubated with the reduced form of nicotinamide-adenine dinucleotide and pyruvate at 37 °C for 15 min and the reaction was stopped by adding NaOH (0.4 mol/L). The activity of LDH was calculated from the absorbance at 440 nm and background absorbance was subtracted from all absorbance measurements.

### Measurement of intracellular ROS

Generation of ROS was monitored by the measurement of superoxide (O_2_^−^) and hydrogen peroxide (H_2_O_2_) using the fluorescent probes dihydroethidium (DHE) and Dichloro-fluorescein diacetate (DCF-DA, Molecular Probes, Eugene, OR, USA). Briefly, cardiomyocytes were seeded and cultured on glass coverslips, treated with CHS and HG separately. After staining for 30 min in the dark with DHE and DCF-DA stains, the cells were washed with PBS and fixed with 4.0% paraformaldehyde (Santa Cruz, USA) in PBS. The glass cover slip was mounted on a glass slide, observed and analyzed using a confocal laser-scanning microscope (Nikon C2 Plus, Tokyo, Japan).

### Measurement of intracellular and mitochondrial calcium

Cytosolic and mitochondrial calcium was estimated by mitochondria impermeant calcium fluorophore, Fluo-3 AM (2 μM) and mitochondria-permeant calcium fluorophore, Rhod-2 AM (2 μM) (Molecular Probes, Eugene, OR, USA), respectively^56^. Cultured H9c2 cells or primary cardiomyocytes grown on glass slides were loaded with Fluo-3 AM or Rhod-2 AM for 30 min after different treatment at 37 °C, fixed with 4% buffered paraformaldehyde, washed 3× with PBS and mounted with antifade on a glass slide for image acquisition using a confocal laser-scanning microscope as described above (Nikon C2 Plus, Tokyo, Japan). Untreated unstained cells served as negative control and untreated fluorophore loaded cells served as control for background correction for confocal experiments.

### Western blot analysis

The cells were harvested and lysed with buffer containing 0.5% SDS, 1% Nonidet P-40, 1% sodium deoxycholate, 150 mM NaCl, 50 mM Tris–HCl (pH 7.5), and protease inhibitors. Cell lysates were centrifuged at 12,000 g for 20 min at 4 °C, and the supernatant was collected and stored at −80 °C. Protein content in the cell extracts was measured using the BCA protein assay kit according to manufacturer’s instructions (Pierce, Thermo Fisher Scientific, UK). Equivalent amounts of protein (30 μg) were loaded and separated on 10% SDS-PAGE gels followed by transferring to a polyvinylidene difluoride (PVDF) membranes (Bio-Rad, Hercules, CA). Nonspecific binding was blocked with a 5% non-fat dried milk for 30 min at 37 °C and then incubated overnight at 4 °C with the following antibodies (Cell Signaling Technologies, MA, USA): ERK1/2 (1:1000), P-ERK1/2 (1:500), Homer1a (1:500), SIRT1 (1:500), Bax (1:1000), Bcl-2 (1:1000), caspase-9 (1:1000), cleaved-caspase-9 (1:1000), caspase-3 (1:1000), cleaved-caspase-3 (1:1000) and β-actin (1:1500). After three washes with Tris-buffered saline (pH 7.2) containing 0.05% Tween 20, membranes were incubated with the secondary antibody at room temperature for 1 h. The immunoreactive bands were detected using the ECL method (Millipore, Bedford, MA). Semi-quantifications were performed with densitometric analysis by Quantity One software (BioRad, USA).

### Real-time qPCR

Total RNA was extracted using the TRIzol Reagent (Invitrogen, Carlsbad, CA). RNA concentrations and purities were determined by measuring the absorbance at 260 nm. First-strand cDNA was generated using SuperScript III first-strand synthesis system as recommended by the manufacturer (Invitrogen, Carlsbad, CA). Reverse transcription was performed using 1 μg of total RNA in 12.5 μl of the solution containing 4 μl 25 mM MgCl_2_, 4 μl AMV reverse transcriptase 5× buffer, 2 μl dNTP, 0.5 μl RNase inhibitor, 1 μl of AMV reverse transcriptase, and 1 μl of oligo dT primer, which were added with nuclease-free water to make a final volume of 20 μl. Reaction system was run at 42 °C for 50 min and 95 °C for 5 min. Primers used for the reactions were purchased from Invitrogen and the sequences of Homer1a: forward, 5′-CGGAATTCTATCTTCAGCACTC-3′, reverse, 5′-CGGAATTCAATGCATTCTGAGC-3′; GAPDH: forward, 5′-ATGTATCCGTTGTGGATCTGAC-3′; reverse, 5′-CCTGCTTCACCACCTTCTTG-3′. Real-time qPCR was carried out in a 20 μl reaction buffer that included a 10 μl of TaqMan Universal PCR Master Mix, 1 μl of primer, and 9 μl of cDNA with the BIO-BAD CFX96 Touch^TM^ Real-Time qPCR system. The GAPDH normalized data are presented as the fold change in gene expression in the treatment group compared with controls.

### siRNA transfection

SIRT1 and ERK1/2-specific short interfering RNA (siRNA) was obtained from Cell Signaling Technology. Homer1a-specific siRNA and scramble control siRNA molecules were chemically synthesized by Shanghai Genechem Company. Transfection was performed using Lipofectamine 2000 (Invitrogen, Carlsbad, CA), according to the manufacturer’s protocol. Following 48 hours transfection, cardiomyocytes were pretreated with CHS and subjected to HG.

### Measurement of mitochondrial membrane potential (MMP)

MMP was measured using the fluorescent dye rhodamine 123 (Rh123, Molecular Probes, Eugene, OR, USA). MMP depolarization resulted in the loss of Rh123 from the mitochondria and a decrease in intracellular fluorescence. Rh123 was added to cultured cells to achieve a final concentration of 10 μM for 30 min at 37 °C after the cells had been treated differently and washed with PBS. The fluorescence was observed by using confocal laser-scanning microscope.

### Flow cytometric analysis of cardiomyocyte apoptosis

The percentage of apoptotic cells measured using the Annexin-V/PI kit for flow cytometry as manufacturer’s instruction (Nanjing Jiancheng Bioengineering Institute, Nanjing jiancheng, China). Cardiomyocytes were washed with PBS, centrifuged at 800 g for 5 min, resuspended in ice-cold PBS, centrifuged at 800 g for 5 min. Cells were re-suspended in binding buffer containing propidium iodide (PI) and FITC-labelled Annexin V and incubated for 30 min at 37 °C in the dark, and analyzed with flow cytometry (FACS Vantage-BD Sciences, USA). The data was analyzed using Cell Quest software for determining the percent of apoptotic cells.

### Assessment of mitochondrial cytochrome c release

To evaluate the subcellular localization of cytochrome c, we used confocal imaging of cells double-labeled with Mitotraker Red CMX Ros (Molecular Probes, Eugene, OR, USA) and a cytochrome c antibody (Cell Signaling Technologies, MA, USA). Briefly, cells with different treatment were incubated with 100 nM Mitotraker Red CMX Ros for 20 min at 37 °C, washed with PBS for three times, and fixed with 4.0% paraformaldehyde in PBS at 37 °C for 10 min. Fixed cells were permeabilized with 0.1% Triton X-100 for 5 min at 4 °C, followed by 30 min incubation at 37 °C in blocking solution (5% nonfat dry milk in PBS) and then primary monoclonal cytochrome c antibody (1:100) for 4 h at 37 °C. After washing, cells were incubated for 1 h in PBS containing FITC-conjugated goat anti-mouse antibody (1:100; Molecular Probes). Cells were then imaged in dual-scan mode on a confocal microscope (Nikon C2 Plus, Tokyo, Japan).

### Statistical analysis

The experiment was performed at least three times, and all values are expressed as means ± standard deviation (SD). Data were compared by one-way ANOVA. p < 0.05 was considered statistically significant.

## Additional Information

**How to cite this article**: Duan, J. *et al*. Chikusetsu saponin IVa confers cardioprotection via SIRT1/ERK1/2 and Homer1a pathway. *Sci. Rep*. **5**, 18123; doi: 10.1038/srep18123 (2015).
